# Antibiotic Resistance: Understanding and Responding to an Emerging Crisis

**DOI:** 10.3201/eid1710.111066

**Published:** 2011-10

**Authors:** Jean Patel

**Affiliations:** Author affiliation: Centers for Disease Control and Prevention, Atlanta, Georgia, USA

**Keywords:** antibiotic resistance, antimicrobial drug resistance, bacteria, response, emerging crisis, Antibiotic resistance: understanding and responding to an emerging crisis, book review

Interest in the problem of antimicrobial drug resistance was once limited to health care workers and academics. Today, the problem is widespread, and patients are acquiring drug-resistant infections for which there may be no active therapeutic agents. Antimicrobial drug–resistant infections, such as methicillin-resistant *Staphylococcus aureus*, have spread beyond health care settings and now cause disease, including life-threatening infections, in community settings.

The goal of Antibiotic Resistance: Understanding and Responding to an Emerging Crisis by Drlica and Perlin is to explain “how human activities contribute to the problem of resistance.” The book is intended for “farmers, hospital administrators, government regulators, health department personnel, pharmaceutical executives, and especially individual users” of antibiotics. The book is written in language that readers with a cursory understanding of biology will understand. Readers with little to no biology background can turn to helpful appendixes in the back of the book, where they will find a concise and clear introduction to the Molecules of Life and Microbial Life Forms, which provide the necessary information to tackle antibiotic resistance concepts.

The subject matter was not short-changed in the authors’ effort to make it more widely accessible. This book addresses resistance problems in all types of infections: bacterial, viral, parasitic, and fungal. Chapters address diverse issues, including antibiotic drug activity, development, and use; resistance mechanisms and transmission; laboratory detection of resistance; and what we can do to avoid drug-resistant pathogens.

The book’s readability is enhanced by the use of text boxes, which provide additional information such as historical antidotes that give depth to the chapter’s topic. For example, in Chapter 3, A Survey of Antibiotics, Box 3-2 recounts how a toxic formulation of sulfa drugs in the late 1937, which killed more than 100 persons, led Congress to pass the Food Drug and Cosmetic Act and to the subsequent creation of the Food and Drug Administration.

In addition, each chapter ends with a Perspective section that concisely positions the chapter’s topic in the larger world of humans, animals, pathogens, and drugs. This book fulfills its intended purpose and will serve as an important resource for anyone looking for greater understanding of antibiotics and the problem of drug resistance.

